# Fragments of Target Cells are Internalized into Retroviral Envelope Protein-Expressing Cells during Cell-Cell Fusion by Endocytosis

**DOI:** 10.3389/fmicb.2015.01552

**Published:** 2016-01-19

**Authors:** Mai Izumida, Haruka Kamiyama, Takashi Suematsu, Eri Honda, Yosuke Koizumi, Kiyoshi Yasui, Hideki Hayashi, Koya Ariyoshi, Yoshinao Kubo

**Affiliations:** ^1^Division of Cytokine Signaling, Graduate School of Biomedical Sciences, Nagasaki UniversityNagasaki, Japan; ^2^Department of Clinical Medicine, Institute of Tropical Medicine, Nagasaki UniversityNagasaki, Japan; ^3^Department of AIDS Research, Institute of Tropical Medicine, Nagasaki UniversityNagasaki, Japan; ^4^Central Electron Microscope Laboratory, Nagasaki University School of MedicineNagasaki, Japan

**Keywords:** endocytosis, retrovirus, envelope, cell-cell fusion, murine leukemia virus, human immunodeficiency virus

## Abstract

Retroviruses enter into host cells by fusion between viral and host cell membranes. Retroviral envelope glycoprotein (Env) induces the membrane fusion, and also mediates cell-cell fusion. There are two types of cell-cell fusions induced by the Env protein. Fusion-from-within is induced by fusion between viral fusogenic Env protein-expressing cells and susceptible cells, and virions induce fusion-from-without by fusion between adjacent cells. Although entry of ecotropic murine leukemia virus (E-MLV) requires host cell endocytosis, the involvement of endocytosis in cell fusion is unclear. By fluorescent microscopic analysis of the fusion-from-within, we found that fragments of target cells are internalized into Env-expressing cells. Treatment of the Env-expressing cells with an endocytosis inhibitor more significantly inhibited the cell fusion than that of the target cells, indicating that endocytosis in Env-expressing cells is required for the cell fusion. The endocytosis inhibitor also attenuated the fusion-from-without. Electron microscopic analysis suggested that the membrane fusion resulting in fusion-from-within initiates in endocytic membrane dents. This study shows that two types of the viral cell fusion both require endocytosis, and provides the cascade of fusion-from-within.

## Introduction

Cell-cell fusion occurs in various physiological and pathological conditions, such as the formations of muscle (Abmayr and Pavlath, 2012) and placenta (Mi et al., 2000), organ repair by stem cells (Rodic et al., 2004), and malignant transformation (Lu and Kang, 2009). Interestingly, syncytiotrophoblasts are formed by endogenous retroviral envelope (Env) proteins called syncytins (Malassiné et al., 2005, 2007). Membrane fusion mechanism in retroviral entry has been well studied. However, cell-cell fusion mechanism by retroviral Env proteins is less characterized. Pathology of many placental abnormalities including eclampsia remains to be elucidated. Some of these disorders may be induced by impaired syncytiotrophoblast formation. Therefore, it is important to resolve cell-cell fusion mechanism induced by the Env protein for identification of placental diseases caused by impaired syncytin functions and for development of new therapeutic approaches against such diseases. Here, we challenged to elucidate the mechanism of cell-cell fusion by Env proteins of ecotropic murine leukemia virus (E-MLV) and human immunodeficiency virus type 1 (HIV-1).

There are two types of cell-cell fusion induced by retroviruses. When fusogenic viral Env protein alone is expressed, the cells fuse with neighboring susceptible cells, called fusion-from-within. On the other hand, when viral particles are inserted into interface between two host cells and simultaneously fuse with the both cells, syncytia are formed, called fusion-from-without.

Membrane fusion activity of the E-MLV Env protein is regulated by its C-terminal 16-amino acid segment called R peptide. The R peptide is cleaved after virion budding. The R peptide-containing Env protein does not induce fusion-from-within, but the R peptide-truncated Env (R-Env) does, showing that the R peptide cleavage after virion release activates the fusogenicity required for the viral entry (Rein et al., 1994; Kubo and Amanuma, 2003). In the case of HIV-1, the precursor Gag protein inhibits the Env-induced cell fusion (Murakami et al., 2004). Therefore, syncytium formation is efficiently induced, when the wild type HIV-1 Env protein alone is expressed in susceptible cells.

E-MLV particles bind to mouse cationic amino acid transporter 1 (mCAT1) as the infection receptor, and then are internalized into endosomes by host cell endocytosis. Endosomal cathepsin proteases are activated by endosome acidification, and digest the viral Env protein to potentiate its membrane fusion activity (Katen et al., 2001; Kumar et al., 2007). The viruses finally enter host cells by fusion between viral envelope and host cell endosome membranes. This viral entry cascade is found not only in the E-MLV infection but also in infections by Ebola virus (Chandran et al., 2005) and SARS coronavirus (Belouzard et al., 2009). In HIV-1 infection, it has been shown that HIV-1 uses the endocytic process as a mean of infection in some circumstances (Miyauchi et al., 2009). However, the mechanistic details of cell-cell fusion induced by retroviral Env proteins are less clear.

Some studies have indicated that virus-cell membrane fusion during viral infection and cell-cell membrane fusion are different. For example, lymphocyte function-associated antigen-1 (LFA-1) regulates HIV-1 mediated-cell fusion but not viral transmission (Pantaleo et al., 1991), and E-MLV Env mutants containing amino acid substitutions at the R peptide cleavage site do not induce infection but mediate syncytium formation in XC cells (Kubo and Amanuma, 2003). Additionally, it has been reported that cellular transformation by the H-Ras oncogene activates the E-MLV virion-induced fusion-from-without but not infection (Wilson et al., 1992), and that actin inhibitors suppress HIV-1 virion-induced fusion-from-without but not viral entry in NP2-derived cells (Kondo et al., 2015).

Using an endocytosis inhibitor and a dominant negative mutant of dynamin, we probed requirement of endocytosis for the retroviral Env-induced fusion-from-within. Because size of an endosome is much smaller than that of a cell, a whole cell cannot be encapsulated into an endosome. To examine how retroviral Env-induced cell fusion uses endocytosis pathway, we performed fluorescence, time-lapse, and electron microscopies. Small fusion pores were observed in membrane dents at the interface of cells. These results suggested that membrane fusion for the syncytium formation initiates in the membrane dents before intracellular endosome vesicles are formed. Additionally, the fluorescence microscopic analysis revealed that fragments of the target cells are internalized into the Env-expressing cells. This result suggested that endocytosis in the Env-expressing cells is important for the cell-cell fusion, whereas that in the target cells is for the viral entry.

It is thought that the fusion-from-without is induced by membrane fusion at cell surface (Kondo et al., 2015). Interestingly, the endocytosis inhibitor attenuated the fusion-from-without induced by E-MLV, suggesting that endocytosis is involved in the fusion-from-without. Finally, we proposed a cascade of the viral Env-induced fusion-from-within.

## Materials and methods

### Cell lines and plasmids

Human embryo kidney (HEK) 293T, human TE671, human HeLa, mouse SC-1, and African green monkey COS7 cells were maintained in our laboratory. TE671 cells expressing mCAT1 (TE671mCAT1) were constructed as already reported (Kamiyama et al., 2011). NP2 cells expressing CD4 and CXCR4 (NP2/CD4/X4) were kindly provided by Dr. H. Hoshino (Soda et al., 1999). These cell lines were cultured in Dulbecco's modified Eagle medium (DMEM) with 8% fetal bovine serum (FBS), 1% penicillin-streptomycin.

The cDNA clone of the E-MLV infection receptor (mCAT1) was kindly provided by Dr. J. M. Cunningham (Albritton et al., 1989). The CD63 cDNA (endosome marker) was molecularly cloned from HeLa cells by RT-PCR with the LA Taq (TaKaRa) and KOD-Plus (Toyobo) DNA polymerases in our laboratory. The MLV Wt Env (R+Env) and R peptide-truncated MLV Env (R-Env) expression plasmids have been shown previously (Kubo and Amanuma, 2003). An expression plasmid encoding C-terminally GFP-tagged CD63 was constructed in our laboratory. HIV-1 Env (JD34) and nuclear localization signal-containing LacZ (nls-LacZ) expression plasmids were kindly provided by Dr. U. Hazan (Dumonceaux et al., 1998) and Dr. L. Chang (Chang et al., 1999), respectively. An expression plasmid encoding the dominant negative mutant of dynamin that inhibits endocytosis was obtained from Dr. S. L. Schmid (Damke et al., 1994).

### Cell fusion assay

Fusion-from-within induced by retroviral Env proteins was measured as follows. HEK293T cells seeded in 3-cm plates were transfected with pcDNA3.1, MLV R-Env, or HIV-1 Env (1 μg) together with nls-LacZ (1 μg) by the Fugene HD transfection reagent (Promega), and incubated for 24–48 h at 37°C. Target cells (TE671mCAT1 cells for the MLV R-Env, and NP2/CD4/X4 cells for the HIV-1 Env) were pretreated with dynasore (100 μM) or CA074Me (40 μM) for 6 h. The transfected and treated cells were co-cultured for 24 h and stained with 5-bromo-4-chloro-3-indoly-D-galactopyranoside (X-Gal). We counted blue nuclei by a light microscope (Leica). The fusion index was calculated as follows: number of blue nuclei in syncytia/total number of blue nuclei × 100. The target and Env-expressing cells were always co-cultured at ratio of 2:1.

To assess the effect of the dynamin dominant negative mutant on fusion-from-within, HEK293T cells seeded in 3-cm plates were transfected with the nls-LacZ (0.5 μg) and Env expression plasmids (0.5 μg) together with the dynamin mutant expression or pcDNA3.1 plasmid (0.5 μg). These cells were washed by PBS, co-cultured with TE671mCAT1, or NP2/CD4/X4 cells 24 h after the transfection, and stained with X-Gal 24 h after the co-culture.

Fusion-from-without induced by E-MLV particles was measured as follows. Target mouse SC-1 cells were seeded onto 12 wells, and pretreated with dynasore (25 and 50 μM) or CA074Me (10 and 20 μM) for 4 h. Undiluted culture supernatants from ecotropic Friend MLV Env-expressing TELCeB6 cells (Kamiyama et al., 2011) were inoculated to the pretreated SC-1 cells. The areas of syncytia were measured by the Image J software 24 h after the inoculation.

### Cell viability assay

HEK293T, TE671mCAT1, SC-1, HeLamCAT1 cells were treated with the inhibitors for 6 h. The cells were collected and treated with trypan blue. Numbers of unstained and stained cells were counted using a counting chamber, and ratios of unstained live cells per total cells were calculated.

### Fluorescence microscopy or video microscopy imaging

For observation by fluorescence microscopy and time lapse analysis, HEK293T cells were cultured in glass slides or glass bottom dishes, and transfected by the Env expression plasmid (0.1 μg) together with GFP or CD63-GFP expression plasmid (0.1 μg). Target cells were stained with the cell tracker orange (TaKaRa) for 1 h. The transfected and stained cells were co-cultured, fixed, and observed under a conforcal fluorescence microscope (Olympus). Time lapse imaging (ASTEC or Keyence) was performed on the live cells.

### Electron microscope

For observation by an electron microscope, HEK293T cells were transfected by the HIV-1 Env plasmid (0.1 μg). The cells were co-cultured with NP2/CD4/X4 cells for 30 min. The co-cultured cells were fixed with 2.5% glutaraldehyde overnight at 4°C. After fixation in osmium tetroxide and ethanol-dehydrated, samples were embedded in epoxy resin. Sections (75–80 nm) were stained with uranyl acetate and then with lead nitrate, and observed using a transmission electron microscope (JEM-1210, JEOL).

## Results

### Endocytosis in env-expressing cells is required for cell fusion contrary to viral infection

**Figure 1:**
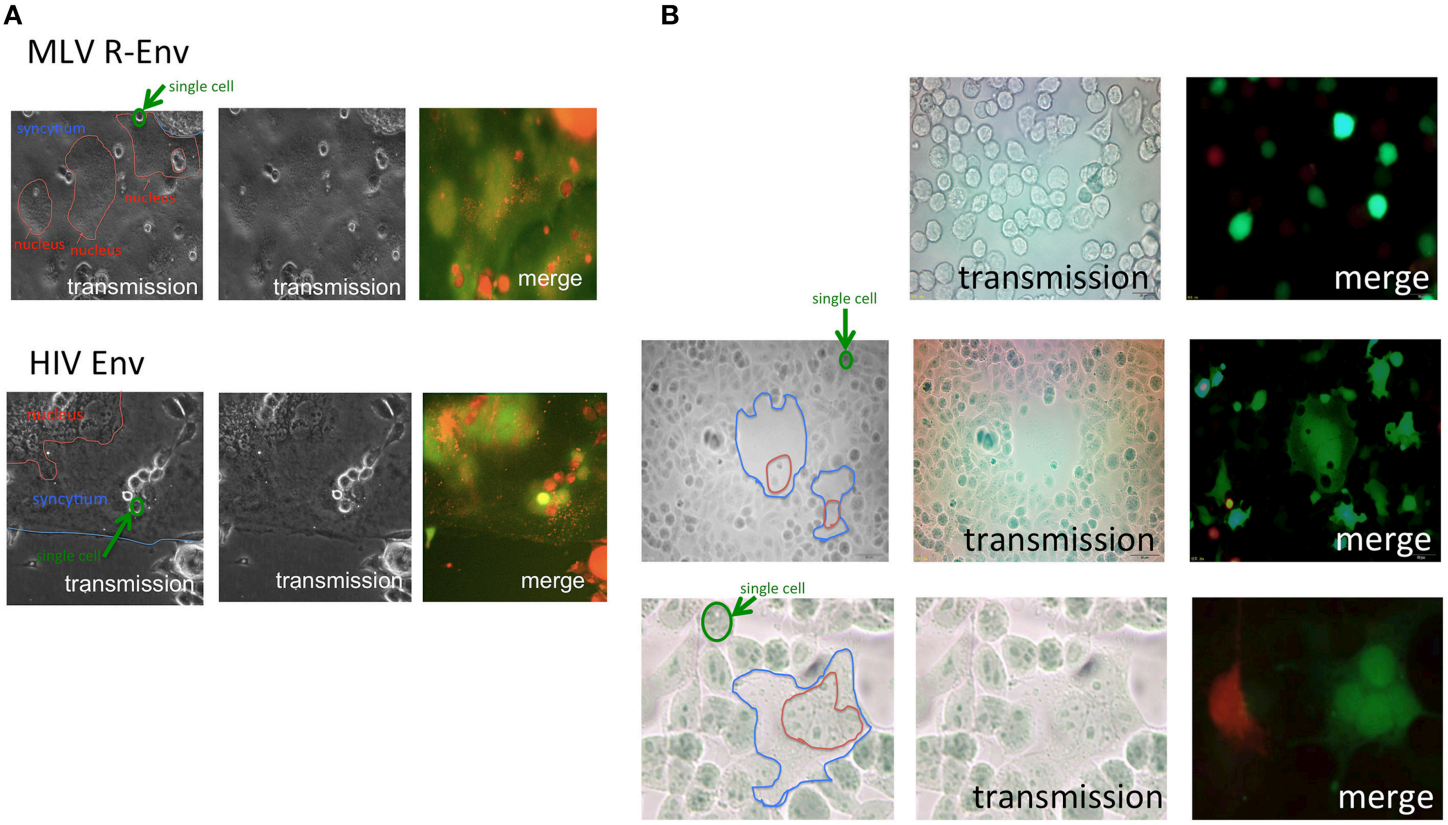
**Target cells are internalized into E-MLV R-Env-expressing cells. (A)** Expression plasmids of the E-MLV R-Env (upper panel) or HIV-1 Env (lower panel) were transfected to HEK293T cells together with the GFP expression plasmid. TE671mCAT1 or NP2/CD4/X4 cells were stained with the cell tracker orange, and co-cultured with the transfected cells. These cells were observed under a fluorescent microscopy. Interfaces of syncytia are indicated by blue lines, and nucleic assemblies are surrounded by red lines. Single cells are surrounded by green line for scale. **(B)** HEK293T cells were transfected with the R+Env and GFP expression plasmids, and co-cultured with cell tracker orange-stained TE671mCAT1 cells (upper panel). HEK293T cells were transfected with the R-Env and GFP expression plasmids, and co-cultured with cell tracker orange-stained TE671 plus unstained TE671mCAT1 cells (middle and lower panels).

Endocytosis of host cells is required for the retroviral infection. To examine whether the cell-cell fusion also requires endocytosis, we investigated syncytia between different fluorescence-labeled Env-expressing and host cells by confocal fluorescence microscopy. The E-MLV R-Env or HIV-1 Env expression plasmid was transfected to HEK293T cells together with a GFP expression plasmid. TE671 cells expressing the E-MLV receptor (TE671mCAT1) were stained with the cell tracker orange, and co-cultured with the transfected cells. Interestingly, many small orange dots under green background were observed in fused cells induced by the E-MLV R-Env (Figure 1A upper panel) and the HIV-1 Env (Figure 1A lower panel). Relatively large orange signals represent unfused cells on syncytia. When 293T cells were transfected with the non-fusogenic R peptide-containing Env expression plasmid together with the GFP expression plasmid, and co-cultured with the cell tracker orange-stained TE671/mCAT1 cells, such orange dots were not detected in the GFP-expressing cells (Figure 1B upper panel). Similarly, when the R-Env-transfected 293T cells were co-cultured with unstained TE671mCAT1 cells and cell tracker orange-stained TE671 cells, small orange dots were not observed in green fused syncytia (Figure 1B middle and lower panels). When unsusceptible orange cells were located near fused cells, the syncytia were not enlarged. These results show that the small orange dots are specifically generated in fused cells. These observation suggested three possibilities; (i) small fragments derived from the target cells are internalized into the Env-expressing cells, (ii) the target cells are fragmented after enclosing the whole target cells by the Env-expressing cells like phagocytosis, or (iii) after the cell fusion, the target cell cytoplasm are not mixed with the Env-expressing cell cytoplasm in the fused cells, and are fragmented.

**Figure 2:**
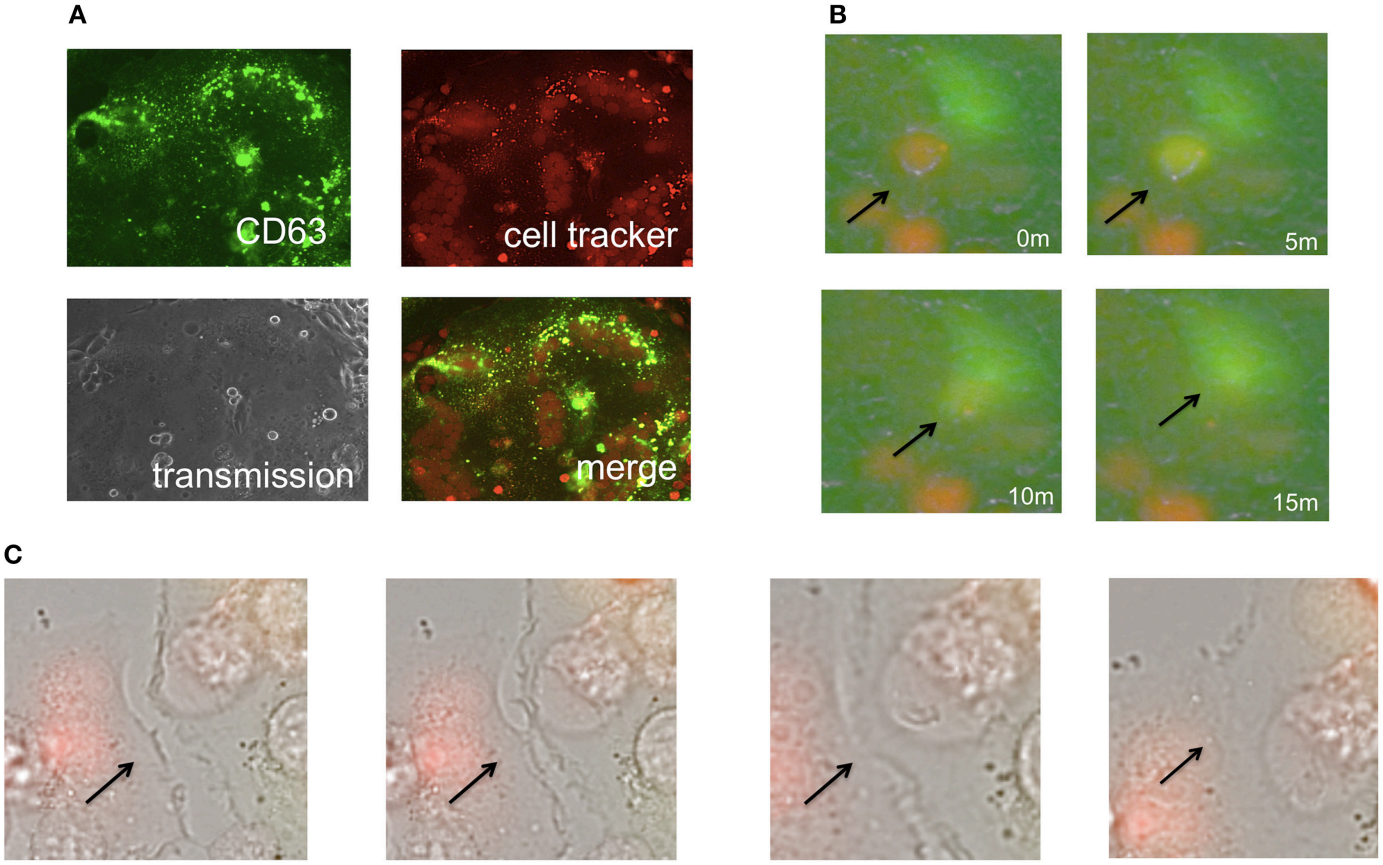
**Phagocytosis is not involved in the cell-cell fusion. (A)** Expression plasmids encoding the R-Env and CD63-GFP were transfected to HEK293T cells. Target cells were stained with the cell tracker orange, and co-cultured with the transfected cells. **(B,C)** HEK293T cells were transfected by R-Env and GFP. mCAT1-expressing TE671 cells were stained with the cell tracker orange. One day after transfection, these cells were co-cultured in glass bottom dish and observed by time-lapse microscopy. Merged pictures of green and red fluorescence images are shown in **(B)**, and those of transmission, green, and red images are shown in **(C)**. Arrows indicate the places where cell fusions occur.

Because size of endosomes is much smaller than that of cells, and because 293T cells do not have an ability of phagocytosis, the second possibility is unlikely. Because the cell tracker orange is a low molecular size compound and more rapidly diffuses than the GFP protein, the third possibility is also unlikely. Thus, the first possibility is most likely, and the small orange dots might represent intracellular vesicles such as endosomes/lysosomes. To assess the hypothesis, the E-MLV R-Env was transfected to HEK293T cells together with a C-terminally GFP tagged CD63 (CD63-GFP) expression plasmid, since CD63 is specifically localized to late endosomes and lysosomes (Pols and Klumperman, 2009). Target cells were stained with the cell tracker orange, and co-cultured with the Env plus CD63-GFP-expressing cells 1–2 days after the transfection. We found that almost all of small orange dots in fused cells were co-located with CD63-GFP signals (Figure 2A). This result indicated that parts of the target cells were encapsulated into late endosomes/lysosomes of the Env-expressing cells during the cell fusion, and strongly supported the first possibility.

### Time-lapse analysis of cell fusion

To analyze cell fusion dynamics, we performed time-lapse imaging analysis of cell-cell fusion induced by the E-MLV R-Env protein. As the result, time course of the cell fusion was as follows. First, a cell tracker orange-stained target cell contacted to an Env-expressing green cell (Figure 2B and Supplementary Video 1). Second, the cell color of the target cells was changed from orange to yellow, indicating that cytoplasm is mixed resulted from the membrane fusion. Third, those cells were merged to form a symcytium. Due to low resolution of the time-lapse fluorescence microscope, orange dots were not observed in fused cells.

Additionally, the time-lapse imaging showed that ruffling structures of cell membranes bound each other, and cell-fusion occurred (Figure 2C). Surprisingly, these cells were already fused cells, indicating that fused cells can fuse with fused cells. These results suggested that membrane fusion during the Env-induced syncytium formation takes place in ruffling membranes, then cytoplasm are mixed by membrane fusion, and finally the cells were merged to form a syncitium. This result also excludes the possibility that phagocytosis is involved in the cell fusion.

### Endocytosis in env-expressing cells is more important for cell fusion than that in target cells

**Figure 3:**
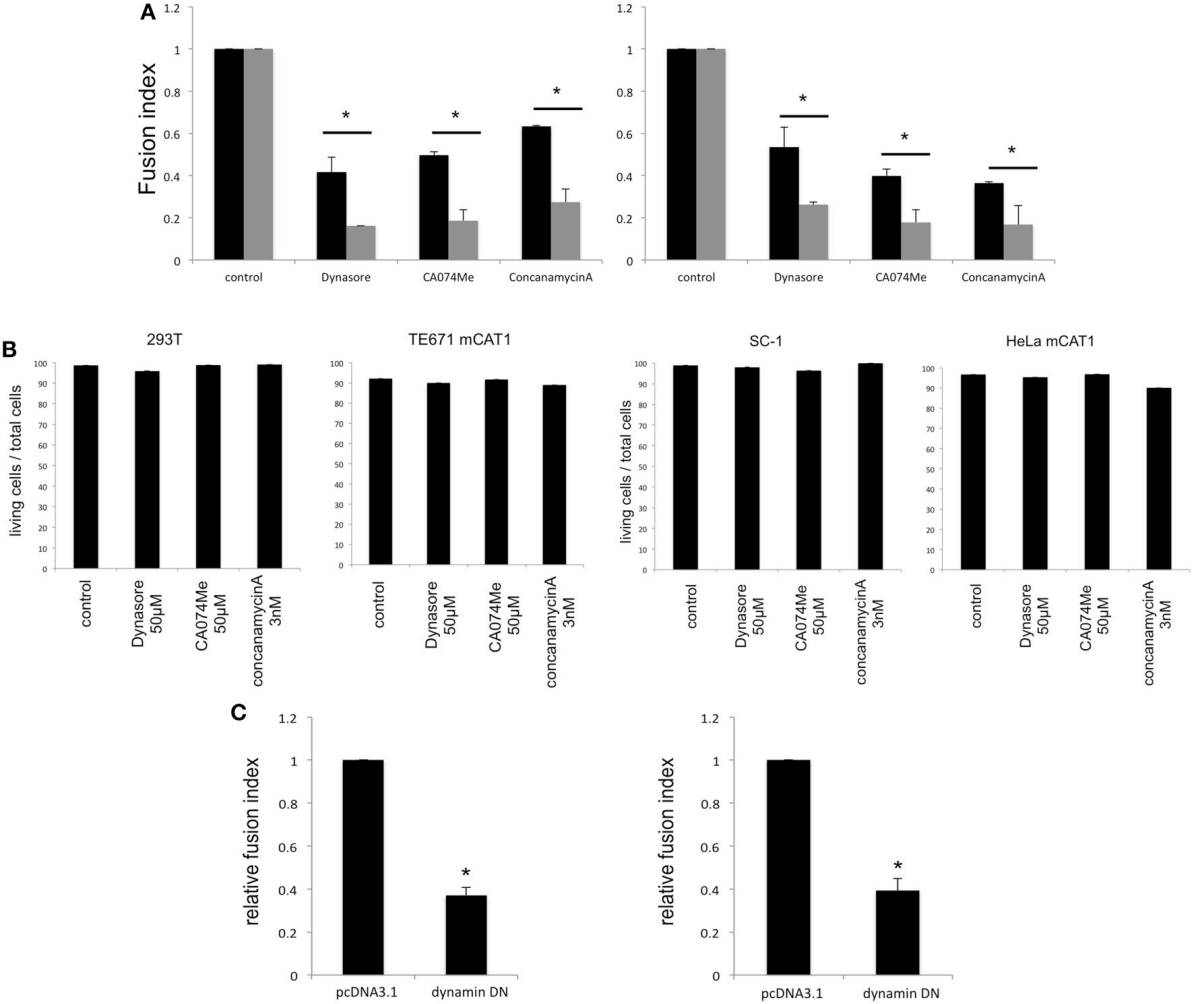
**Endocytosis in Env-expressing cells is required for the cell-cell fusion. (A)** The E-MLV R-Env (left panel) or HIV-1 Env (right panel) was transfected to HEK293T cells together with the nls-LacZ expression plasmid. The transfected HEK293T cells were co-culture with TE671mCAT1 or NP2/CD4/X4 cells pretreated with indicated inhibitor (black bars). The transfected cells were treated with indicated inhibitors, and were co-cultured with TE671mCAT1 or NP2/CD4/X4 cells (gray bar). This experiment was repeated three times. Fusion indexes in the control cells were always set to 1, and relative fusion indexes ± SD are shown. Asterisks indicate statistically significant differences between treatments of the Env-expressing or target cells. **(B)** HEK293T, TE671mCAT1, SC-1, and HeLamCAT1 cells were treated with indicated inhibitor. Ratios (%) of live cells per total cells are indicated. This experiment was repeated three times. **(C)** HEK293T cells were transfected with the MLV R-Env (left panel) or HIV-1 Env (right panel) expression plsmid together with the nls-LacZ expression plasmid. The cells were also simultaneously transfected with the dynamin dominant negative mutant expression plasmid or pcDNA3.1. This experiment was repeated three times. Fusion indexes in the control cells were always set to 1, and relative fusion indexes ± SD are shown. Asterisks indicate statistically significant differences between transfection with pcDNA3.1 and dynamin mutant.

To confirm whether fragments of the target cells are internalized into the Env-expressing cells by endocytosis, the Env-expressing or target cells were treated with an inhibitor of endocytosis (dynasore), endosomal cathepsin B (CA074Me), or endosome acidification (concanamycin A). Syncytium formation induced by the E-MLV R-Env (Figure 3A left panel) or by the HIV-1 Env (Figure 3A right panel) was more efficiently inhibited when the Env-expressing cells were treated than when the target cells were treated, showing that the syncytium formation requires endocytosis, cathepsin B, and endosome acidification in the Env-expressing cells. Because the treatment with each inhibitor did not affect cell viability (Figure 3B), the suppression of cell fusion by the inhibitors should not result from unexpected effects of the inhibitors. Additionally, when the E-MLV R-Env (Figure 3C left panel) or HIV-1 Env (Figure 3C right panel) expression plasmid was transfected to HEK293T cells together with a dominant negative mutant of dynamin, cell-cell fusion was inhibited. These results suggest that endocytosis in the Env-expressing cells are required for the cell-cell fusion. Unfortunately, the target cells stably expressing the dynamin dominant negative mutant were not obtained. When the target cells were transiently transfection with the dynamin mutant, syncytium formation was not affected due to the mutant expression in only several % of transfected cells. Thus, the impact of dynamin mutant expressed in target cells on the cell-cell fusion could not be analyzed.

### Electron microscopy analysis

**Figure 4:**
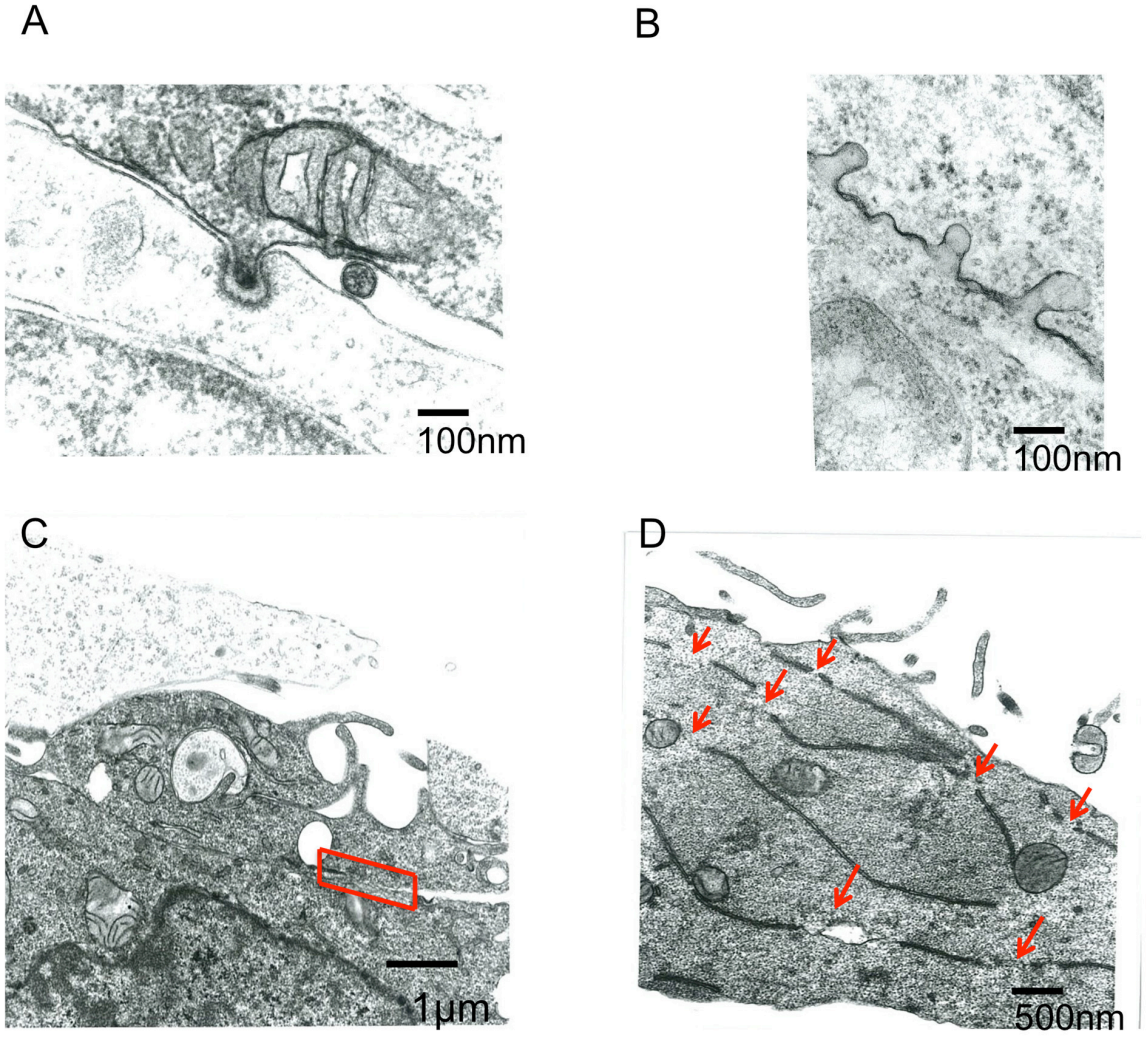
**Electron microscope observation of fused cells**. HIV-1 Env-transfected 293T and NP2/CD4/X4 cells were co-cultured. The cells were fixed 30 min after the co-culture. **(A,B)** Membrane dents in the interface of cells were observed. **(C)** Plasma membrane indicated by red box was disappeared. **(D)** Long and narrow compartments were observed in syncytia. Red arrows indicate gaps between the long and narrow compartments.

**Figure 5:**
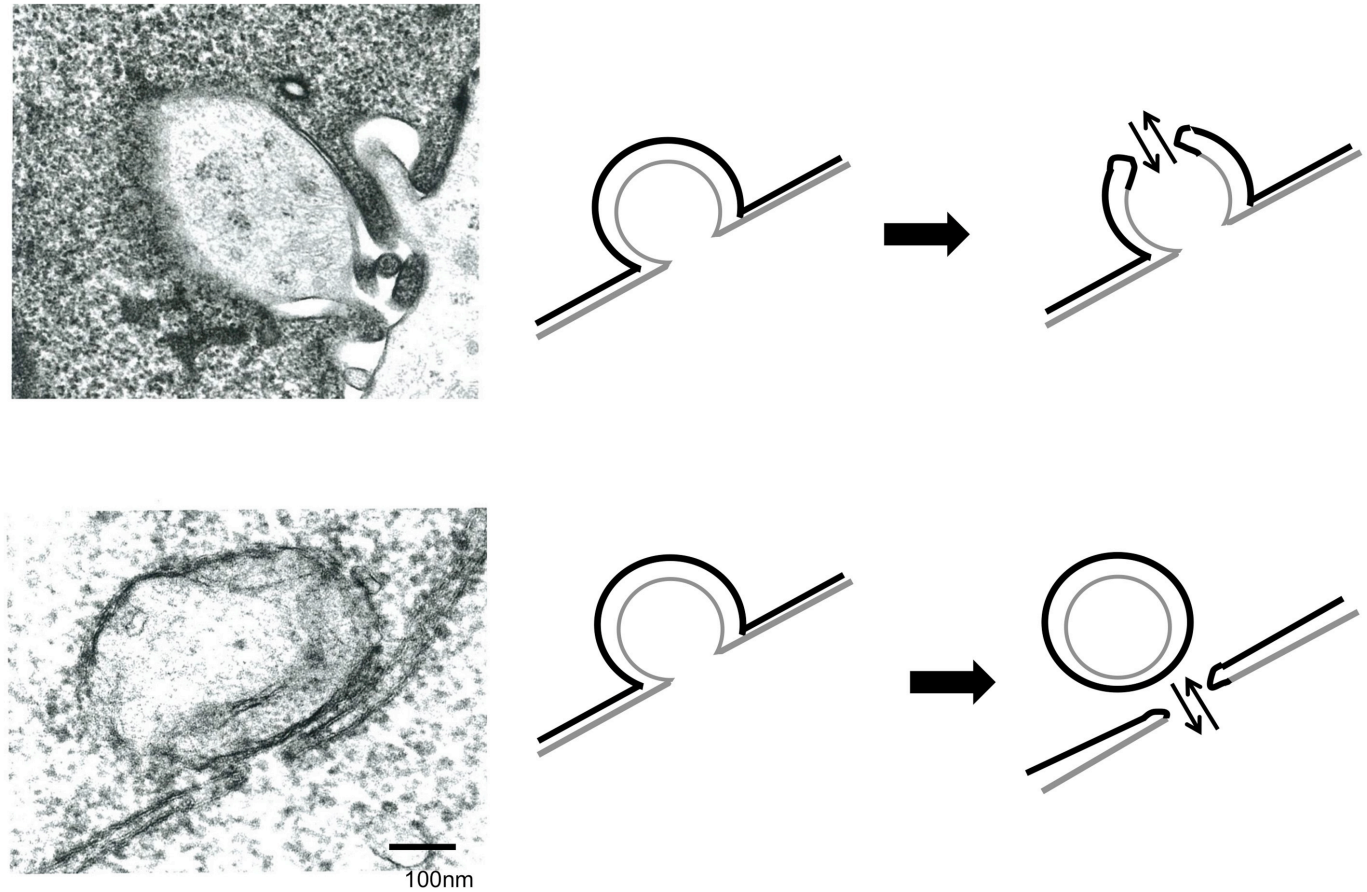
**Membrane fusion occurs at membrane dents**. HIV Env-transfected and NP2/CD4/X4 cells were co-cultured. The cells were observed under an electron microscopy.

The above results showed that endocytosis in the Env-expressing cells is required for the retroviral Env-induced cell-cell fusion. To know how endocytosis is involved in the cell fusion, electron microscopic observation at the cell-cell contact area was performed. It was observed that small parts of a cell were invaginated into another cell at the cell-cell contact area like endocytosis (Figures 4A,B). Two types of fused membrane were observed by the electron microscopy; (i) a relatively wide area of plasma membrane at the cell-cell contact region disappeared by membrane fusion (Figure 4C, red box), and (ii) a small membrane region at a tip of a membrane dent disappeared by membrane fusion (Figure 5 upper panel). Because in the former type of fused membrane, relatively wide membrane area always disappeared, it was thought that their fusion pores were already enlarged. In contrast, the fusion pores observed in the membrane dents were smaller and not enlarged yet, suggesting that the fusion pores in the membrane dents are nascent and newly formed. These results suggested that the membrane fusion starts at membrane dents.

Additionally, long and narrow membrane vesicles were detected in cytoplasm of fused cells (Figure 4D). These compartments should be derived from plasma membrane remaining between the membrane fusion sites when more than two membrane fusions occurred at a cell-cell contact area. Thus, membrane fusions should occur at gaps of the long and narrow compartments (Figure 4D, red arrows). Interestingly, a round vesicle was observed at a gap of long and narrow compartments (Figure 5 lower panel). It was thought that the vesicle is formed by membrane fusion at the root of invaginating membrane, which results in cytoplasm mixing. This result also supported the conclusion that the membrane fusion starts at the membrane dent. Such intracellular vesicles were not observed at many of the nicks of the long and narrow compartments (Figure 4D). The nicks without vesicles might be formed by the membrane fusion at a tip of a membrane dent mentioned above (Figure 5 upper panel). Thus, it was suggested that the membrane fusion at a tip of a membrane dent more preferentially occurs than that at a root.

### Endocytosis promotes fusion-from-without

**Figure 6:**
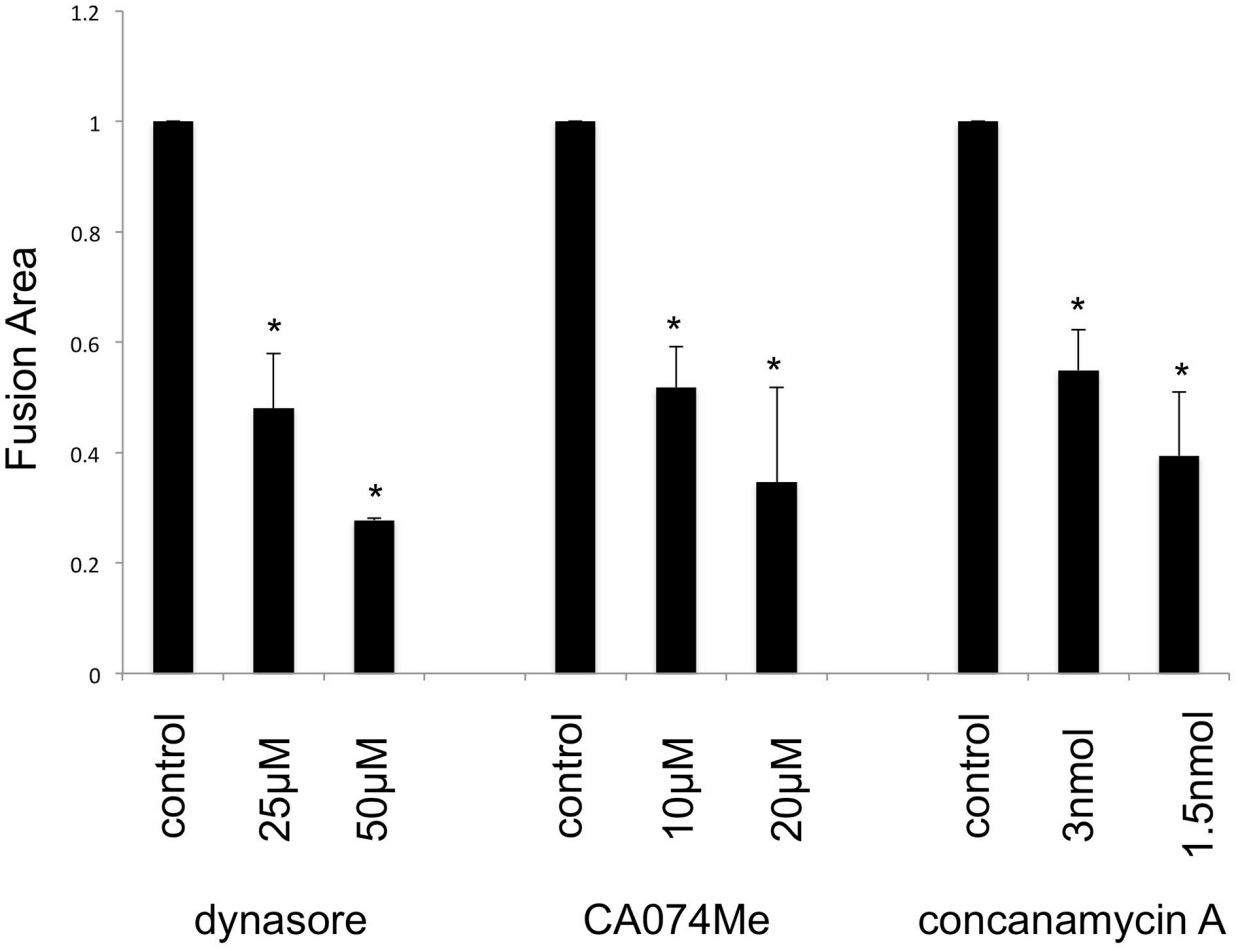
**Fusion-from-without requires endocytosis**. Mouse SC-1 cells were pretreated with dynasore, CA074Me, or concanamycin A, and were inoculated by E-MLV. Sizes of fused cell areas are measured. This experiment was repeated three times. Sizes of fused cell areas in control cells are always set to 1, and relative values ± SD are indicated. Asterisks indicate statistically significant differences between sizes of fused areas in solvent- and inhibitor-treated cells.

It is thought that the fusion-from-without is induced by membrane fusion at cell surface, and does not required endocytosis. To confirm this issue, target cells were pretreated with dynasore, CA074Me, or concanamycin A, and were inoculated with E-MLV to form fusion-from-without. Contrast to the previous theory, these inhibitors significantly suppressed the fusion-from-without (Figure 6). This result showed that endocytosis, cathepsin B, and endosome acidification are required for the fusion-from-without.

## Discussion

In this study, our fluorescence and electron microscopic observations showed that fragments of the target cells were internalized into the Env-expressing cells. In addition, the treatment of the Env-expressing cells with an endocytosis inhibitor reduced syncytium formation more effectively than that of target cells. These results indicated that endocytosis in the Env-expressing cells triggers the cell-cell fusion. Target cells treated with the inhibitor also reduced syncytium formation, because the target cells become Env-expressing cells after the fusion with the Env-expressing cells.

**Figure 7:**
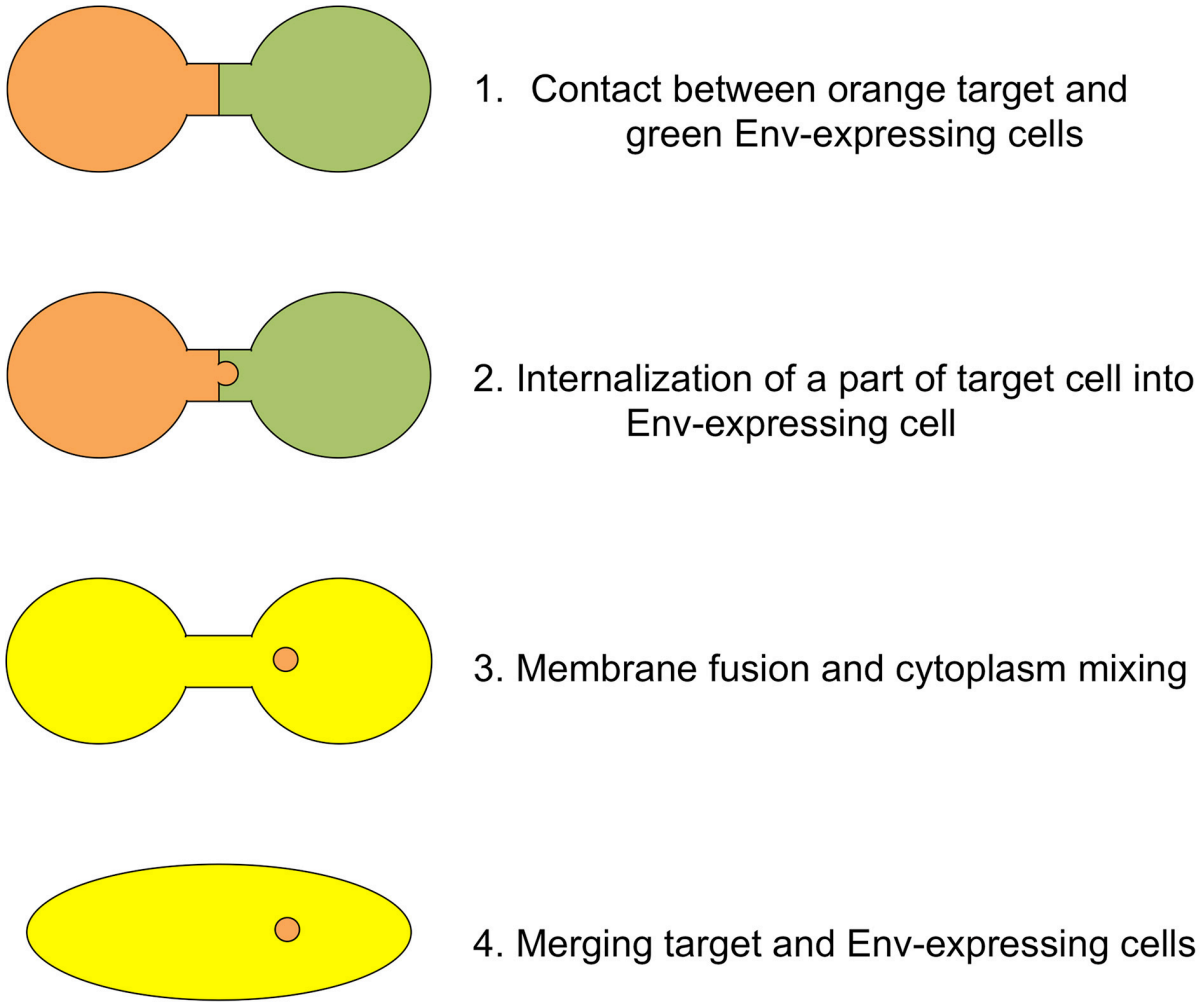
**Schematic presentation of cell-cell fusion induced by retroviral Env protein**. Orange and green cells indicate target and Env-expressing cells, respectively. When cytoplasm of these cells are mixed, the color of cell is changed to yellow.

Based on our results, we propose the cascade of retroviral Env-induced cell-cell fusion as follows (Figure 7). First, a target cell (orange) contacts with an Env-expressing cell (green) through ruffling membranes. Second, parts of the target cell are inserted into the Env-expressing cell at the contact region by endocytosis. Third, membrane fusion occurs at the endocytic membrane dent, and cytoplasm of the target cells are mixed with that of the Env-expressing cells (yellow). Vesicles containing the target cell cytoplasm (orange) remain in the fused cell. Fourth, cells are merged completely to form a syncytium. It was thought that a little proportion of invaginating endosomes participates for cell-cell fusion, because many small vesicles containing the target cytoplasm were detected in the fused cells.

We showed here two patterns of membrane fusion. One membrane fusion occurs in the tip of membrane dent, and the other occurs at the root of membrane dent (Figure 5). The membrane dents might be induced by endocytosis. Because dynamin is involved in pinching endosomes off and does not affect the membrane dent formation, dynasore can inhibit the membrane fusion at a root of membrane dent, but not that at a tip. The membrane fusion at a root of membrane dent might be induced by dynamin-mediated scission of both the target and Env-expressing cell membranes. The membrane fusion at a tip of membrane dent might result from activation of the Env fusogenicity by acidification of spaces between target and Env-expressing cells. Our study cannot exclude the possibility that the membrane fusion resulting in cell-cell fusion occurs other places than membrane dents. It was difficult to find the membrane dents with fusion pores, suggesting that the nascent fusion pore is unstable and easily enlarged. Further study is required to elucidate the mechanism by which endocytosis promotes the cell fusion.

The treatment of Env-expressing cells with the cathepsin B inhibitor also suppressed the cell-cell fusion induced by the Env proteins of E-MLV and HIV-1. Although the E-MLV infection requires cathepsin B, the inhibitor does not attenuate HIV-1 Env-mediated infection (Yoshii et al., 2011). How is cathepsin B involved in the HIV-1 Env-induced cell fusion? Further study is needed to understand this issue.

Interestingly, it was observed that a fused cell fuses with a fused cell. Because fused cells express the Env protein, it is thought that the infection receptor proteins in the fused cells are occupied by the Env proteins, and the fused cells are not susceptible to the Env-induced cell fusion. Nascent syncytia might partially express the infection receptors that are not occupied by the Env proteins yet, and fuse with the Env-expressing cells. Alternatively, when the expression level of Env protein is low, the free infection receptor molecules still remain on cell surface of the fused cells, and can fuse with Env-expressing cells.

This study found that the fusion-from-without also requires endocytosis. When a viral particle is internalized into a target cell by endocytosis, another cell bound to the virion may be also internalized into the cell. Further study is needed to understand the mechanism of fusion-from-without.

In summary, this study found that the fusion-from-within induced by the E-MLV or HIV-1 Env protein requires endocytosis in the Env-expressing cells, although the viral entry requires endocytosis in target cells. The fusion pore is initiated in endocytic membrane dents. The fusion-from-without also requires endocytosis.

## Author contributions

MI, HK, EH, Y. Koizumi, KY, and Y. Kubo performed experiments. TS helps the experiment of electron microscopy. MI, HH, KA, and Y. Kubo analyzed the data. MI and Y. Kubo wrote the manuscript.

### Conflict of interest statement

The authors declare that the research was conducted in the absence of any commercial or financial relationships that could be construed as a potential conflict of interest.

## Supplementary material

Supplementary Video 1 **Time-lapse video microscopic observation of syncytium formation**. HEK293T cells were transfected by the R-Env and GFP expression plasmids. TE671/mCAT1 cells were stained with the cell tracker orange. One day after transfection, these cells were co-cultured in glass bottom dish and observed by time-lapse microscopy.
